# Serum superoxide dismutase level is a potential biomarker of disease prognosis in patients with hemorrhagic fever with renal syndrome caused by the Hantaan virus

**DOI:** 10.1186/s12879-022-07394-3

**Published:** 2022-05-10

**Authors:** Zhen Tian, Naijuan Yao, Yuchao Wu, Fei Wang, Yingren Zhao

**Affiliations:** 1 Department of Infectious Diseases, The First Affiliated Hospital of Xi’an Jiaotong University, 277 West Yanta Road, Xi’an city, 710061 Shaanxi China; 2 Department of Ultrasound, The First Affiliated Hospital of Xi’an Jiaotong University, 277 West Yanta Road, Xi’an city, 710061 Shaanxi China

**Keywords:** Oxidative stress, Inflammation, Hemorrhagic fever with renal syndrome, Prediction

## Abstract

**Background:**

Hemorrhagic fever with renal syndrome (HFRS) is a disease with increased systemic inflammation and a high fatality rate. Oxidative stress is crucial for inflammation in the pathogeneses of various diseases. We aimed to identify biomarkers of oxidative stress that may assess the severity and disease outcomes of patients with HFRS.

**Methods:**

Between January 2015 and September 2018, we analyzed a retrospective cohort of 149 HFRS patients and 30 healthy individuals. Serum levels of SOD were measured using an ELISA commercial kit, and survival analysis was carried out using the Kaplan–Meier method.

**Results:**

Patients with HFRS had significantly lower serum SOD levels compared with healthy controls (108.40 ± 2.47 U/mL vs 164.23 ± 3.82 U/mL, P < 0.01). SOD levels in patients were lower at acute than at convalescent stage (108.40 ± 2.47 U/mL vs 138.27 ± 2.87 U/mL, P < 0.01), and in severe and critical patients than in moderate and mild patients (89.63 ± 2.38 U/mL vs 122.53 ± 3.18 U/mL, P < 0.01). A serum level of SOD < 88.6 U/mL at admission was associated with a significant increase in mortality risk in HFRS patients.

**Conclusion:**

Our results indicate that serum levels of SOD measured at admission can be used to assess disease severity and assign patients into high- and low-risk groups. SOD can be considered a novel biomarker of severity and outcomes in patients with HFRS.

**Supplementary Information:**

The online version contains supplementary material available at 10.1186/s12879-022-07394-3.

## Background

Hantaviruses are a group of enveloped single-stranded negative RNA viruses, that can cause hantavirus cardiopulmonary syndrome in America and hantavirus hemorrhagic fever with renal syndrome (HFRS) in Eurasia[1, 2]. HFRS is a febrile disease characterized by hypotension, hemorrhage, and kidney injury. There are at least four types of HFRS-causing hantaviruses, including Hantaan, Seoul, Puumala, and Dobrava[3, 4]. The phases of HFRS clinical presentation include febrile, hypotensive, oliguric, diuretic, and convalescent phases[5]. The predominant etiological factors of HFRS in China are the Seoul virus and Hantaan virus, which are linked with mild, moderate, severe, and critical forms[6].

Despite vaccination, approximately 10,000 cases of HFRS are reported annually in mainland China, with a mortality rate of 0.1–15%[7]. Xi’an City, the capital of Shaanxi province, is one of the most affected areas in China[8]. Apart from monitoring and supportive management, there are currently few available medications; therefore, early detection and diagnosis are critical. Although several hypotheses have been proposed to explain the pathophysiology of HFRS, there are still no reliable laboratory criteria for predicting disease severity and mortality. We hypothesized that a biomarker representing the disease etiology might be used to assess the severity and prognosis of HFRS.

Excessive inflammation has been linked to a variety of human diseases, including HFRS[9, 10]. When excessively activated, inflammation serves as a double-edged sword, leading to both beneficial antimicrobial responses and tissue damage[11]. Increased levels of pro-inflammatory cytokines such as TNF-α, IL-6, IL-8, and IL-10 have been observed in HFRS patients in earlier studies[9, 12]. IL-1β, a significant endogenous pyrogen and a marker of active inflammation, has also been reported to be higher in the serum of HFRS patients. By causing fever and an inflammatory environment, IL-1β prevents invasion of the Hantaan virus; moreover, IL-1β increases vascular permeability via IL-1β receptors[13].

Previous research has demonstrated that oxidative stress plays an important role in inflammation, since reactive oxygen species (ROS) have been found to activate the NLRP3 inflammasome, resulting in the production of active IL-1β in various diseases[14]. Furthermore, Hantavirus (HTNV) causes the formation of the NLRP3 inflammasome in THP-1 cells via ROS, which might be the cause of high IL-1β levels in HFRS patients, according to a recent study [15]. Several laboratory indicators, including some inflammatory markers, have been utilized to determine the severity and clinical outcomes of HFRS since it is a life-threatening disease [16, 17]. However, none of these markers are sensitive or specific enough, as they concentrate on the clinical phenomenon rather than on the disease pathogenesis. Since it is assumed that HFRS is characterized by excessive inflammation driven by ROS, we hypothesized that an oxidative stress biomarker could be used to predict the disease severity and outcomes.

Superoxide dismutase (SOD) is a key endogenous antioxidant enzyme that protects cells from intracellular and extracellular oxidative damage. It reduces the harmful effects of ROS by converting damaging superoxide to hydrogen peroxide[18]. In individuals with acute liver failure (ALF), serum SOD level was demonstrated to be a reliable predictor of disease severity [19]. Furthermore, given that HFRS is linked to excessive inflammation and oxidative stress, in this study we focused on serum SOD, to determine whether serum SOD level is a reliable predictor of the disease severity and outcomes.

## Methods

### Patients

A total of 149 individuals with typical HFRS at the First Affiliated Hospital of Xi’an Jiaotong University from January 2015 to September 2018 were included. This retrospective study was conducted in accordance with the Declaration of Helsinki. Written informed consent was obtained from each patient and/or legal guardian of deceased patients, and the study was approved by the Research Ethics Committee of the First Affiliated Hospital of Xi’an Jiaotong University. During the same period, 30 age- and sex-matched healthy participants were recruited as controls.

The identification of IgM and IgG antibodies specific to HTNV in acute phase serum specimens by ELISA was used to diagnose HFRS. We used IgG/IgM capture ELISA kits and analyzed them via a multifunctional autoanalyzer (BIORAD-Model 680, USA)[20]. All the patients were divided into four groups based on the following clinical classification of HFRS based on the diagnostic criteria from the Prevention and Treatment Strategy of HFRS by the Ministry of Health, People’s Republic of China as described elsewhere[16]: 1) mild, defined as patients who had kidney injury without oliguria and hypotension; 2) moderate, defined as patients who had uremia, effusion (bulbar conjunctiva), hypotension, hemorrhage (skin and mucous membranes), and acute kidney injury (AKI) with typical oliguria; 3) severe, defined as patients who had severe uremia, effusion (bulbar conjunctiva and either peritoneum or pleura), hemorrhage (skin and mucous membranes), hypotension and AKI with oliguria (urine output of 50–500 mL/day) for ≤ 5 days or anuria (urine output of < 100 mL/day) for ≤ 2 days; 4) critical, defined as severe patients who had one or more of the following additional complications: refractory shock (≥ 2 days), visceral hemorrhage, heart failure, pulmonary edema, brain edema, severe secondary infection, and severe AKI with oliguria (urine output of 50–500 mL/day) for > 5 days or anuria (urine output of < 100 mL/day) for > 2 days.

The so-called acute stage of the disease is commonly characterized as the period encompassing febrile, hypotensive, and oliguric phases, while the convalescent stage includes diuretic and convalescent phages[21]. The outcome was defined as death or survival during the hospital stay, and for the next 28 days after discharge.

### Estimation of serum SOD

Blood samples were collected at admission, and then stored at – 80 ℃ within 2 h of collection. A commercially available kit (#EIASODC, Thermo Fisher Scientific, Waltham, MA, USA) was used to detect serum SOD levels in line with the manufacturer’s protocol. The sensitivity of the assay was 0.044 U/mL, and the samples and standards were run in duplicate.

Data management and laboratory parameters.

Demographic and clinical data of HFRS patients, including age, gender at the time of the assessment, hospital stay, blood transfusion, continuous renal replacement therapy (CRRT), and HFRS-related complications such as hemorrhage, secondary bacterial infection, hepatic injury, sepsis, multiple organ dysfunction syndrome (MODS), and arrhythmia were collected. Serum WBC, PLT, APTT, INR, AST, ALT, Cre, and BUN were measured at hospital admission.

### Statistical methods

Means and standard deviations (SDs) were used to present the findings. Demographic characteristics were compared using a chi-square test or Fisher's exact test for categorical data, and Wilcoxon rank-sum test for continuous variables. Receiver operating characteristic (ROC) curves were used to examine the predictor value of SOD for disease prognosis. The maximum of the sum of sensitivity and specificity was used to define cutoffs for continuous variables. Log-rank tests were used to compare Kaplan–Meier survival curves to 28 days post-discharge. SPSS version 16.0 software (IBM Corporation, Somers, NY, USA) was used to analyze the data. When P < 0.05, differences were considered statistically significant.

## Results

### Clinical typing and demographic characteristics of HFRS patients

A total of 149 HFRS patients were included in the present research. According to the HFRS clinical categorization criteria, among the enrolled patients, there were 64 mild, 21 moderate, 6 severe, and 58 critical patients. Moreover, of the 58 critical HFRS patients, 46 survived and 12 died with a hospital mortality rate of 20.68%. The clinical baseline characteristics of HFRS patients of different clinical types and controls are shown in Table1.

**Table 1:** Demographics and clinical data at admission in the patients with HFRS of different clinical types and healthy control

**Variables**	Mild (n = 64)	Moderate (n = 21)	Sever (n = 6)	Critical (n = 58)	Control (n = 30)
Age, year	42.98 ± 15.57	42.19 ± 13.75	42.83 ± 12.48	43.26 ± 18.94	42.54 ± 8.65
Gender, M/F	48/16	14/7	6/0	46/12	23/7
HFRS complication					
Hemorrhage, n (%)	26 (40.63)	9 (42.86)	4 (66.67)	41 (70.69)	
Bacterial infection	35 (54.69)	11 (52.38)	3 (50)	35 (60.34)	
Hepatic injury, n (%)	28 (43.75)	10 (47.62)	4 (66.67)	36 (62.07)	
Sepsis, n (%)	0	0	1 (16.67)	10 (17.24)	
MODS, n (%)	0	0	0	7 (12.07)	
Arrhythmia, n (%)	2 (3.13)	1 (4.76)	1 (16.67)	9 (15.52)	
Blood transfusion, n (%)	10 (15.63)	3 (14.28)	3 (50)	35 (60.34)	
CRRT, n (%)	0	0	0	31 (53.45)	
Hospital days	10 (3–27)	10 (5–28)	13 (5–36)	14 (5–47)	
Number of deaths n (%)	0	0	0	12 (20.68)	

*MODS* multiple organ dysfunction syndrome, *CRRT* continuous renal replacement therapy

### Levels of SOD in patients with HFRS

**Fig. 1:**
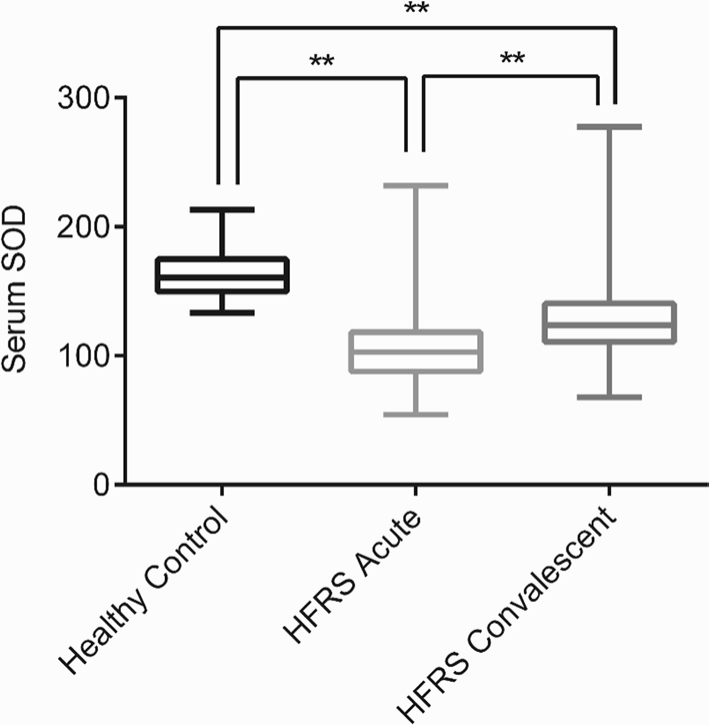
Serum SOD levels in HFRS patients. Comparison of serum SOD in healthy controls and patients with HFRS at acute and convalescent stage. **P < 0.01

SOD levels in both acute and convalescent stage patients were substantially lower than those in healthy controls (acute: 108.40 ± 2.47 U/mL vs 164.23 ± 3.82 U/mL, P < 0.01; convalescent: 138.27 ± 2.87 U/mL vs 164.2 ± 3.82 U/mL, P < 0.01), while SOD levels in the acute stage were lower than those in the convalescent stage (Fig. 1). We then focused on the initial blood samples acquired from HFRS patients during admission in the acute stage. We examined the effects of age and gender on SOD concentration. We found that 76.5% of HFRS patients were male and 23.5% were female. The age range of patients with HFRS ranged from 10 to 70 years. The concentration of SOD in patients with HFRS did not significantly differ among age groups or genders (supplement data, S1 Fig).

### Levels of SOD are associated with disease severity in patients with HFRS

**Fig. 2:**
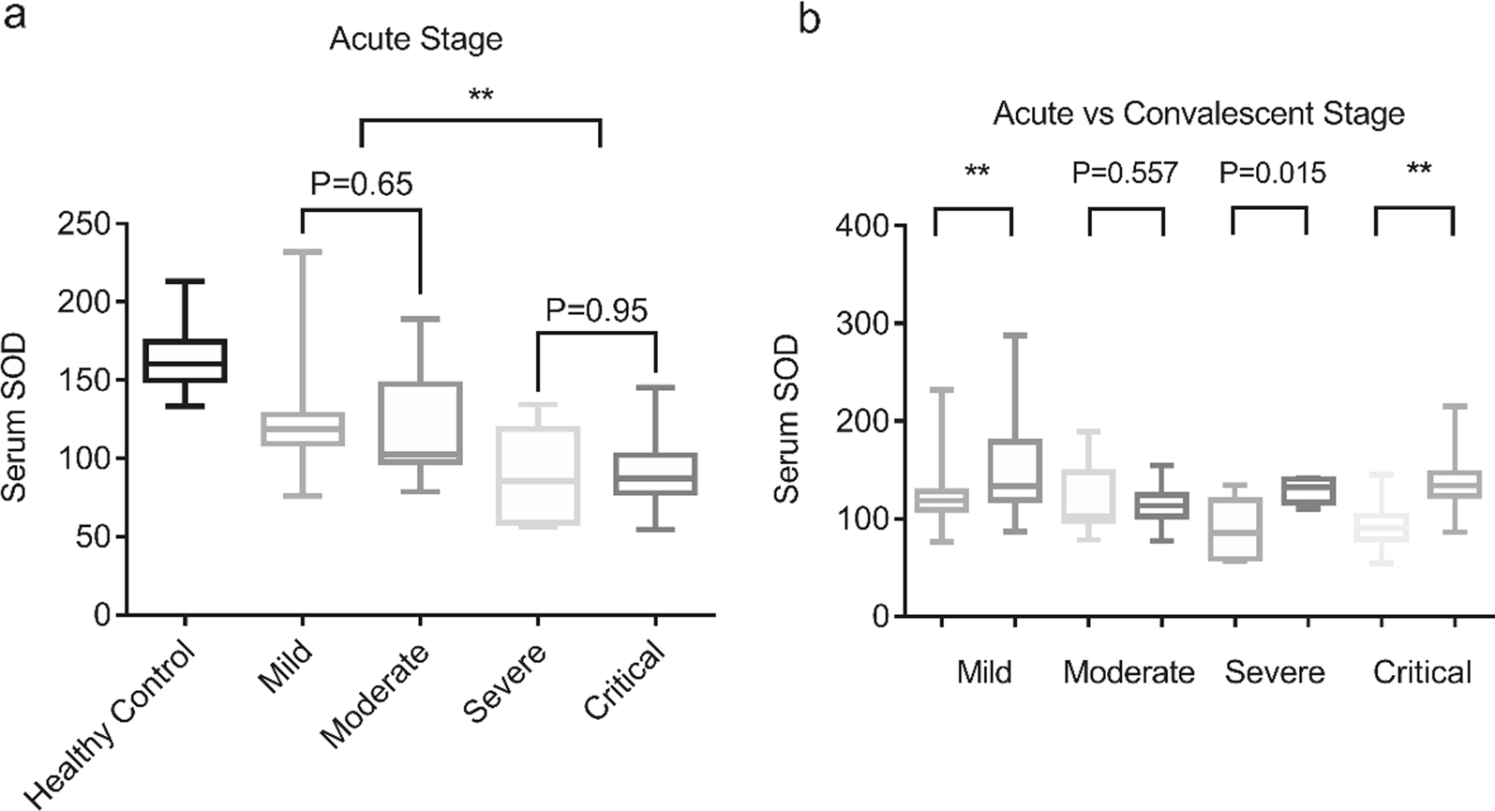
Serum SOD levels in HFRS patients in different groups. **a** Comparison of serum SOD in healthy controls and patients with HFRS according to the clinical course of the disease; **b** Comparison of serum SOD in patients with HFRS at acute and convalescent stage in mild, moderate, severe and critical groups. **P < 0.01

The median serum concentration of SOD in healthy individuals was 1.6-times higher than that in HFRS patients. The serum levels of SOD were then compared among HFRS patients in different groups. Serum SOD levels were considerably lower in the critical group or the severe group of HFRS patients than in the moderate or mild groups, indicating that serum SOD levels may be linked to disease severity in HFRS patients. No significant differences in SOD levels were found between mild and moderate forms (123.37 ± 3.46 U/mL vs 119.98 ± 7.56 U/mL, P = 0.65) or between severe and critical forms (89.13 ± 12.69 U/mL vs 89.68 ± 2.33 U/mL, P = 0.95) (Fig. 2a). With the disease remission, serum SOD levels significantly increased in the mild, severe, and critical groups (Fig. 2b).

### Serum SOD levels are associated with mortality in HFRS patients

**Fig. 3:**
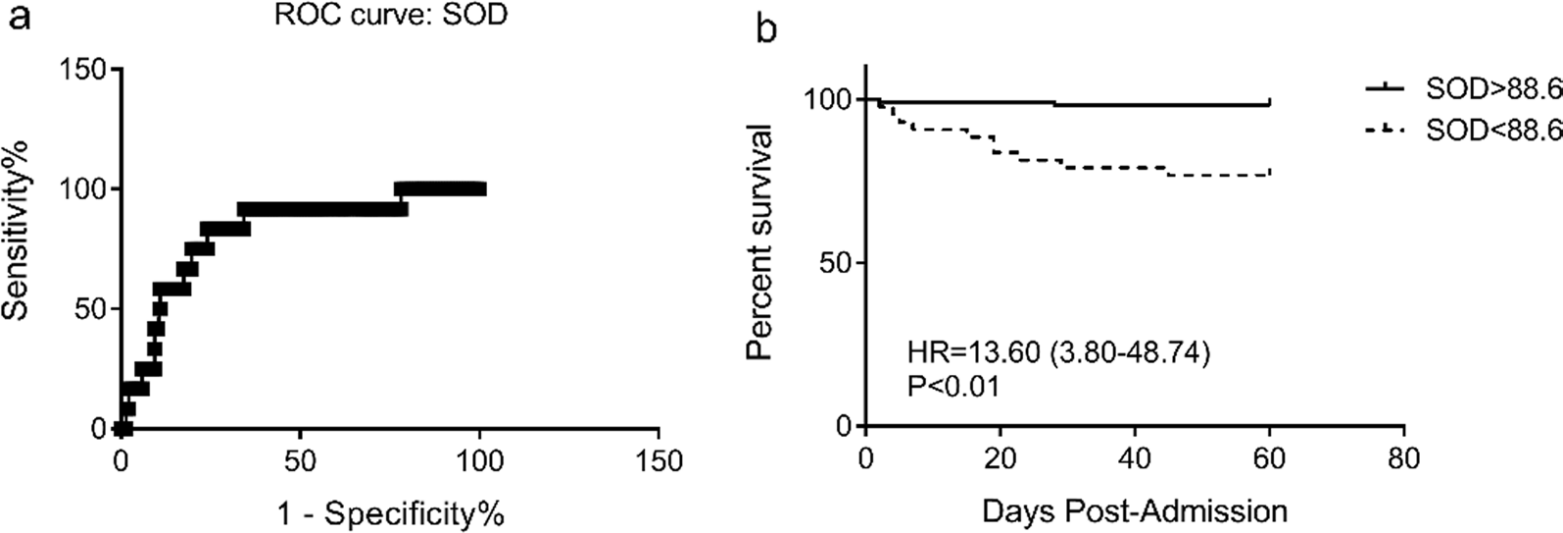
Kaplan–Meier analysis for survival according to admission SOD level. **a** ROC curve of serum SOD; **b** Survival of HFRS patients according to serum SOD level

In the current cohort, among 58 patients were who classified as critical, 12 patients died. The greatest sensitivity and specificity for SOD as a predictor of death in critical HFRS patients was 88.6 U/mL, according to ROC analysis. The Kaplan–Meier analysis showed that HFRS patients with serum SOD levels < 88.6 U/mL had an increased risk of death (P < 0.01) (Fig. 3).

### Laboratory parameters in the patients during the acute stage

The levels of WBC, APTT, AST, ALT, Cre, and BUN were higher or longer than the reference values (P < 0.01), while the PLT count was lower than the reference value (P < 0.05) (Table 2). Compared with patients in the mild group and the moderate group, patients in the severe group and the critical group had lower SOD levels and higher APTT, INR and Cre levels (P < 0.05) (Table 3). Moreover, compared with the survivors, non-survivors have lower SOD levels and more elevated APTT, INR, AST, ALT, and BUN levels (P < 0.05) (Table 4).

**Table 2:** Laboratory data at admission in the patients with HFRS of different clinical types and healthy control

Variables	Mild (n = 64)	Moderate (n = 21)	Sever (n = 6)	Critical (n = 58)	Control (n = 30)
Parameter					
WBC (1 × 10^9^/L)	13.52 ± 18.83	13.93 ± 4.34	13.85 ± 3.95	16.78 ± 12.10	5.68 ± 1.54
PLT (1 × 10^9^/L)	68.25 ± 46.47	42.62 ± 24.61	51.33 ± 35.52	51.55 ± 51.81	226.0 ± 38.54
APTT (s)	41.99 ± 9.21	52.29 ± 16.85	50.23 ± 15.08	57.73 ± 29.88	30.19 ± 3.16
INR	1.10 ± 0.15	1.07 ± 0.08	1.10 ± 0.11	1.18 ± 0.31	1.21 ± 0.19
AST (u/L)	88.78 ± 102.8	99.50 ± 86.33	94.79 ± 65.33	338.8 ± 1243.1	20.56 ± 14.33
ALT (u/L)	86.76 ± 140.9	57.56 ± 54.09	65.39 ± 55.34	129.7 ± 439.0	23.32 ± 15.33
Cre (μmol/L)	229.8 ± 207.6	306.4 ± 151.8	318.3 ± 296.1	332.1 ± 209.1	55.67 ± 12.64
BUN (mmol/L)	15.26 ± 11.14	18.44 ± 9.31	13.19 ± 8.43	18.51 ± 10.73	5.67 ± 3.56
SOD (U/mL)	123.4 ± 3.46	120.0 ± 7.56	89.13 ± 12.69	89.68 ± 2.33	164.2 ± 3.82

*WBC* white blood cell count, *PLT* platelet, *APTT* activated partial thromboplastin time, *INR* international normalized ratio, *AST* aspartate aminotransferase, *ALT* alanine aminotransferase, *Cre* creatinine, *BUN* blood urea nitrogen, *SOD* superoxide dismutase

**Table 3:** Laboratory parameters in patients with HFRS during the acute stage (severity)

Parameter	Mild & moderate	Severe & critical	*P *value
WBC (1 × 10^9^/L)	13.62 ± 16.48	16.51 ± 11.61	0.24
PLT (1 × 10^9^/L)	61.92 ± 43.56	52.71 ± 50.51	0.18
APTT (s)	44.54 ± 12.39	56.98 ± 28.83	< 0.01
INR	1.09 ± 0.13	1.17 ± 0.29	0.038
AST (u/L)	91.43 ± 99.11	315.9 ± 1185.7	0.087
ALT (u/L)	79.54 ± 125.8	123.6 ± 418.6	0.36
Cre (μmol/L)	248.8 ± 198.0	330.8 ± 218.8	0.019
BUN (mmol/L)	16.05 ± 10.80	18.01 ± 10.65	0.27
SOD (U/mL)	122.5 ± 3.18	89.63 ± 2.38	< 0.01

**Table 4:** Laboratory parameters in patients with HFRS during the acute stage (survival)

Parameter	Survivors	non-Survivors	*P* -value
WBC (1 × 10^9^/L)	14.31 ± 14.52	21.10 ± 18.56	0.14
PLT (1 × 10^9^/L)	59.69 ± 47.98	32.00 ± 19.68	0.051
APTT (s)	46.65 ± 14.13	84.03 ± 47.04	< 0.01
INR	1.10 ± 0.18	1.39 ± 0.36	< 0.01
AST (u/L)	88.30 ± 84.47	1324.5 ± 2497.0	< 0.01
ALT (u/L)	67.15 ± 102.1	456.2 ± 896.5	< 0.01
Cre (μmol/L)	277.1 ± 209.2	362.3 ± 217.5	0.18
BUN (mmol/L)	16.30 ± 10.43	23.59 ± 12.35	0.025
SOD (U/mL)	110.7 ± 2.57	82.54 ± 4.63	< 0.01

### ROC and Kaplan–Meier survival analysis

**Fig. 4:**
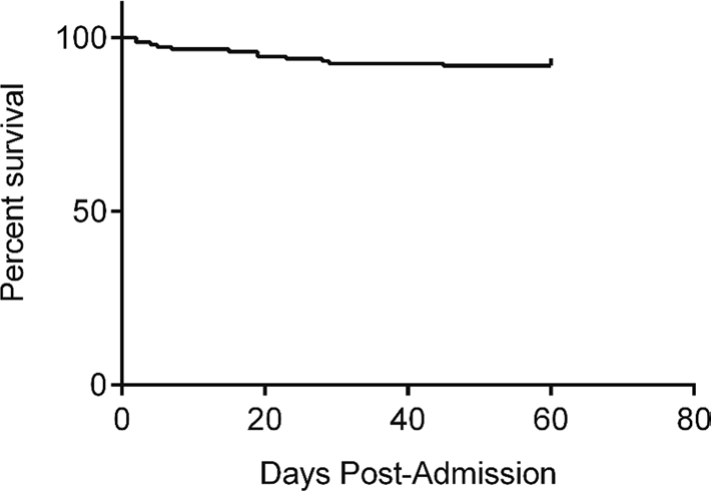
Kaplan–Meier analysis for survival based on the total survival time in the period from the onset of illness to discharge or death in HFRS patients

In HFRS patients, Kaplan–Meier analysis was used to determine the cumulative proportion of survivors and the 60-day death rate (Fig. 4).

We identified the potential risk factors in HFRS patients by using univariate and multivariate Cox regression analyses. As shown in Table 5, PLT (HR = 0.957, 95% CI: 0.921–0.995, P < 0.05), APTT (HR = 1.052, 95% CI: 1.022–1.083, P < 0.01), INR (HR = 33.315, 95% CI: 3.784–293.327, P < 0.01), AST (HR = 1.004, 95% CI: 1.001–1.007, P < 0.01), ALT (HR = 1.004, 95% CI: 1.001–1.006, P < 0.01), BUN (HR = 1.055, 95% CI: 1.005–1.108, P < 0.05) and SOD (HR = 0.946, 95% CI: 0.915–0.979, P < 0.01) were significantly associated with mortality in HFRS patients. Therefore, we performed a forward multivariate analysis. The results revealed that BUN (HR = 1.133, 95% CI: 1.032–1.243, P < 0.01), and SOD (HR = 0.918, 95% CI: 0.859–0.981, P < 0.01) were independent risk factors for mortality in HFRS patients.

**Table 5:** Uni-and multivariate logistic analysis of prognosis factors associated with survival in patients with HBV-ACLF

	Univariate			Multivariate		
	HR	95% CI	*P*	HR	95% CI	*P*
Age (yr)	1.000	0.966–1.036	0.980			
Sex(M/F)	1.710	0.482–6.059	0.406			
WBC (1 × 10^9^/L)	1.017	0.992–1.043	0.183			
PLT (1 × 10^9^/L)	0.957	0.921–0.995	0.027	0.970	0.914–1.029	0.308
APTT (s)	1.052	1.022–1.083	< 0.01	1.059	0.997–1.125	0.061
INR	33.315	3.784–293.327	< 0.01	0.274	0–152.232	0.688
AST (u/L)	1.004	1.001–1.007	< 0.01	1.019	0.996–1.043	0.105
ALT (u/L)	1.004	1.001–1.006	< 0.01	0.951	0.892–1.015	0.123
Cre (μmol/L)	1.002	0.999–1.004	0.188			
BUN (mmol/L)	1.055	1.005–1.108	0.031	1.133	1.032–1.243	< 0.01
SOD (U/mL)	0.946	0.915–0.979	< 0.01	0.918	0.859–0.981	< 0.01

ROC and area under curve (AUC) were analyzed to investigate the predictive value of BUN and SOD for prognosis in HFRS patients. AUC of BUN was much lower than that of SOD, suggesting that SOD is a better marker for predicting mortality in HFRS patients (Table 6).

**Table 6:** Predictive values for prognosis on laboratory parameters in patients with HFRS

**Parameter**	AUC	*P*-value	Cut-off	Sensitivity	Specificity	95% CI for AUC	
						Lower	Upper
BUN	0.6755	< 0.01	15.79	56.93	58.33	51.48	83.62
SOD	0.8178	< 0.01	88.6	83.33	78.10	70.41	93.15

## Discussion

HFRS is an acute viral disease caused by the Hantaan virus and is most endemic in China. Hantaviruses are a family of viruses spread mainly by rodents and may cause a variety of disease syndromes in humans all over the world. Currently, hantavirus pulmonary syndrome (HPS) can be lethal in the USA, and HFRS can be lethal in Eurasia. To avoid a fatal outcome, it is critical to estimate the severity of the disease in early the phases. Several researchers have attempted to uncover predictive biomarkers for HFRS to assess the disease severity and prognosis, but none of these markers were specific and sufficient enough given that they did not focus on the pathogenesis of HFRS. We aimed to identify a marker reflecting disease pathogenesis and predicting the prognosis.

In the present study, we analyzed the changes in serum SOD levels in HFRS patients and found that age and gender had no effect on SOD levels in HFRS patients. We showed that serum SOD level was a possible prediction biomarker of HFRS. Our data revealed significantly lower serum SOD levels in HFRS patients than in healthy individuals. The serum SOD level was also linked to the disease severity, considering that SOD levels were lower in severe and critical patients than in moderate and mild patients. We previously discovered that cytokine and chemokine levels fluctuate during HFRS and tend to recover during the remission stage. In the present study, we found that SOD levels were higher in convalescent patients than in acute patients, indicating the prognostic value of serum SOD level in assessing the disease severity in HFRS patients. Next, we assessed the prognostic value of serum SOD level and found that HFRS patients with SOD < 88.6 U/mL had a considerably higher risk of death. Serum SOD level can be easily measured using an ELISA kit, and may serve as an independent predictor of mortality in HFRS patients. Taken together, very early testing of the serum SOD level may predict the HFRS outcomes.

SOD is an endogenous antioxidant enzyme that converts poisonous superoxide into hydrogen peroxide, thereby limiting the harmful effects of ROS[22]. A previous study showed that influenza A virus infection led to autophagic degradation of SOD, thereby contributing to increased ROS generation in alveolar epithelial cells[23]. According to our findings, the pathogenesis of HFRS is likely influenced by oxidative stress. Importantly, HTNV has been shown to promote ROS production[24]; thus, we assumed that during the pathogenesis of HFRS, HTNV may also lead to autophagy-mediated SOD degradation, which would contribute to the accumulation of ROS. Since endothelial cells are the main targets of HTNV, damaged/dead endothelial cells may also cause increased oxidative stress, thereby contributing to ROS accumulation during HFRS.

According to earlier studies, ROS are necessary for NLRP3 inflammasome activation. ROS then initiates the assembly of inflammasomes, activation of caspase-1, and caspase-1-dependent IL-1β secretion. Excessive inflammation occurs in many human illnesses, including HFRS, and contributes to both helpful antimicrobial responses and cell death when it is overactive. Endothelial cells and monocytes are the principal targets of HTNV. When activated by HTNV, these cells release pro-inflammatory and inflammatory molecules, which are thought to play a key role in the pathogenesis of HFRS. In previous studies, increased levels of pro-inflammatory cytokines such as TNF-α, IL-6, IL-8, and IL-10, and inflammatory cytokine IL-1β were reported in HFRS patients. IL-1β then induces fever and creates an inflammatory environment to prevent HTNV invasion, and causes increased vascular permeability by activating IL-1 receptors[25], which plays an important role in HFRS pathogenesis. Moreover, recent studies have shown that IL-1β can lead to decreased SOD activity and promote ROS production[26]. Increased oxidative stress stimulates the inflammasome, resulting in the death of even more endothelial cells. The relationship among endothelial cells, oxidative stress, and inflammation then creates a vicious cycle.

## Conclusions

Our findings were derived from a large cohort over 3 years, demonstrating the generalizability of our findings. However, the current study's limitations include those common to retrospective analysis, including potential biases such as selection bias, and all of the patients in the current study were enrolled at a single center. Additionally, only 12 patients in this study died, which might have affected the results of the ROC analysis. Moreover, the mechanism by which HTNV leads to decreased SOD levels was not investigated. Thus, we assumed that HTNV may lead to autophagy-mediated SOD degradation during the pathogenesis of HFRS, but this hypothesis needs more investigation.

The present study indicated that oxidative stress was likely involved in the pathogenesis of HFRS. Very early testing of serum SOD could probably be used for predicting the disease severity and mortality. In addition, the development of strategies to modulate oxidative stress might provide insight into the pathogenic mechanisms and could be a potential target for treating HFRS patients.

## Supplementary Information

**Additional file 1. Fig S1.** Serum SOD levels in HFRS patietns. SOD concentration in HFRS patients according to gender and age distribution.

## Data Availability

The datasets generated during and analyzed during the current study are not publicly available due to HFRS caused by Hantavirus is a notifiable disease in China and local government did not agree to make the datasets public, but the datasets are available from the corresponding author on reasonable request.

## Abbreviations

HFRS: Hemorrhagic fever with renal syndrome
ROS: Reactive oxygen specie
HTNV: Hantavirus
SOD: Superoxide dismutase
ALF: Acute liver failure
AKI: Acute kidney injury
CRRT: Continuous renal replacement therapy
MODS: Multiple organ dysfunction syndrome
ROC: Receiver operating characteristic
AUC: Area under curve
