# Supplementary figures and images for: Serum superoxide dismutase level is a potential biomarker of disease prognosis in patients with hemorrhagic fever with renal syndrome caused by the Hantaan virus
Source: BMC Infect Dis. 2022 May 10;22:446. doi: 10.1186/s12879-022-07394-3 (PMC9087930; doi:10.1186/s12879-022-07394-3)

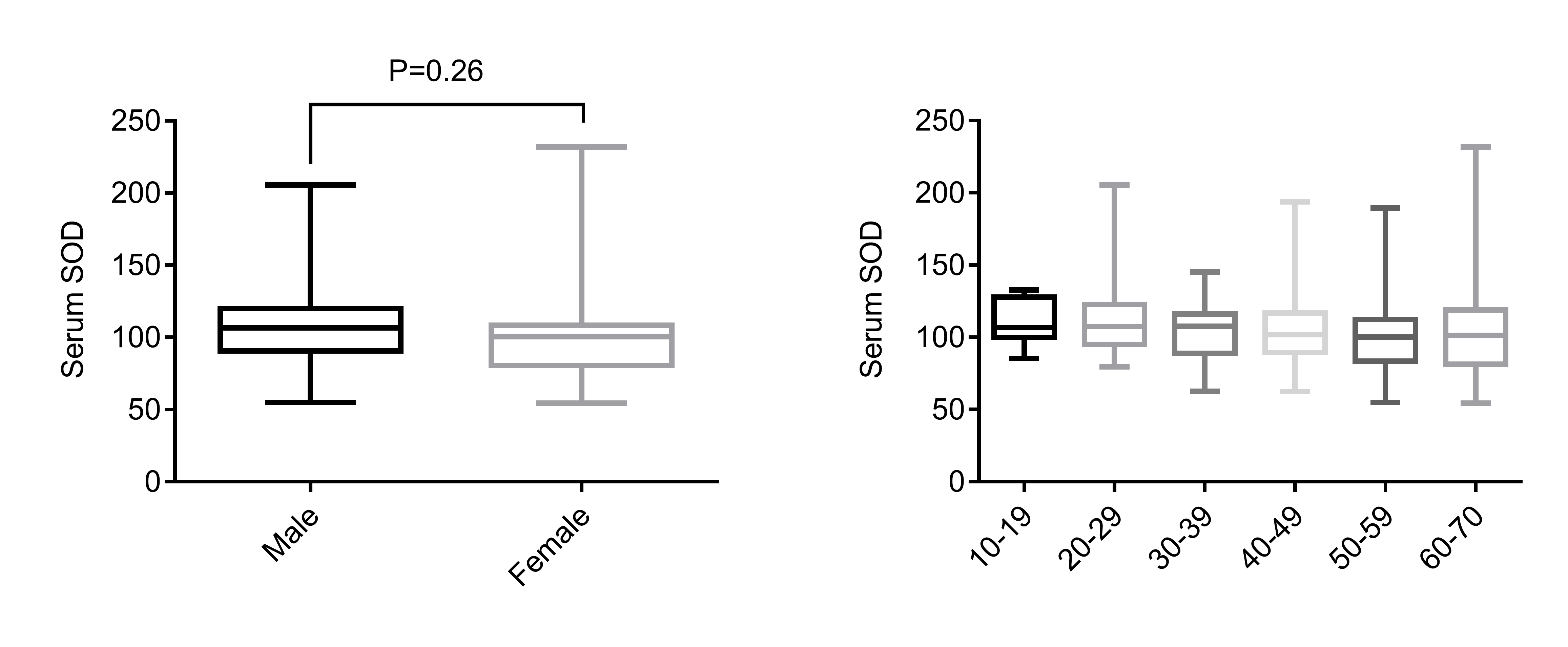

Supplement: Supplementary file 1 — Additional file 1. Fig S1. Serum SOD levels in HFRS patietns. SOD concentration in HFRS patients according to gender and age distribution. [file 12879_2022_7394_MOESM1_ESM.tif]
